# A clinical and pharmacokinetic study of the combination of etravirine plus raltegravir in HIV patients with expanded intolerance or resistance

**DOI:** 10.7448/IAS.17.4.19804

**Published:** 2014-11-02

**Authors:** Sara Bañón, Ana Moreno, Carmen Quereda, Cristina Gomez, Alberto Diaz de Santiago, María J Perez-Elías, Santiago Moreno, Jose Luis Casado

**Affiliations:** Infectious Diseases, Ramon y Cajal Hospital, Madrid, Spain

## Abstract

**Introduction:**

The combination of etravirine (ETR) plus raltegravir (RAL) could be an option for HIV patients with resistance, intolerance or important interactions with other drugs. However, there are few data on the efficacy, safety and pharmacokinetics of this dual therapy, taking into account the effect of HCV co-infection or the possible induction of ETR in the drug metabolism of RAL.

**Material and Methods:**

Cohort study of HIV patients initiating ETR plus RAL as dual therapy. Plasma trough levels of RAL were measured by LC/MS after at least one month on therapy.

**Results:**

A total of 25 patients have been included in this combination since 2009. Mean age was 46 years, 72% were male, and 20 patients (80%) had HCV co-infection (seven patients with fibrosis 3–4). Median nadir CD4+ count was 109 (60–209), and 21 patients had an HIV RNA level below 50 copies/mL. Median time on previous therapy was 473 months (IQR, 395–570), and reasons for this dual therapy was toxicity/intolerance in 19, and interactions in nine (two chemotherapy, three DAAs, two methadone, two other). After a median follow up of 722 days (473–1088: 53.3 patients-year), there were no cases of blips or virological failure. Six patients (24%) discontinued therapy after more than 1.5 year on therapy, in four cases due to lost follow up and in two due to simplification after finishing the reason for interaction. There were no cases of liver toxicity, and only two patients increased slightly transaminases values (grade 1 and 2). Total cholesterol and triglycerides levels decrease significantly after initiation (TC, from 182 to 165 at one year; p=0.01; TG from 185 to 143 mg/dL; p=0.01). CT/HDL ratio decreases from 4.35 to 4.28 after six months. Geometric mean plasma trough level of RAL was 166 ng/mL (IQR, 40–249) and only one patient (6%) was below the *in vitro* IC50 of the wild type.

**Conclusions:**

The combination of ETR plus RAL as dual therapy is effective and safe in patients with expanded intolerance or interactions. There are no significant pharmacokinetic interactions between both drugs.

